# Why Do Women Not Use Antenatal Services in Low- and Middle-Income Countries? A Meta-Synthesis of Qualitative Studies

**DOI:** 10.1371/journal.pmed.1001373

**Published:** 2013-01-22

**Authors:** Kenneth Finlayson, Soo Downe

**Affiliations:** Research into Childbirth and Health Unit, School of Health, University of Central Lancashire, Preston, United Kingdom; South African Medical Research Council, South Africa

## Abstract

**Background:**

Almost 50% of women in low- and middle-income countries (LMICs) don't receive adequate antenatal care. Women's views can offer important insights into this problem. Qualitative studies exploring inadequate use of antenatal services have been undertaken in a range of countries, but the findings are not easily transferable. We aimed to inform the development of future antenatal care programmes through a synthesis of findings in all relevant qualitative studies.

**Methods and Findings:**

Using a predetermined search strategy, we identified robust qualitative studies reporting on the views and experiences of women in LMICs who received inadequate antenatal care. We used meta-ethnographic techniques to generate themes and a line-of-argument synthesis. We derived policy-relevant hypotheses from the findings. We included 21 papers representing the views of more than 1,230 women from 15 countries. Three key themes were identified: “pregnancy as socially risky and physiologically healthy”, “resource use and survival in conditions of extreme poverty”, and “not getting it right the first time”. The line-of-argument synthesis describes a dissonance between programme design and cultural contexts that may restrict access and discourage return visits. We hypothesize that centralised, risk-focused antenatal care programmes may be at odds with the resources, beliefs, and experiences of pregnant women who underuse antenatal services.

**Conclusions:**

Our findings suggest that there may be a misalignment between current antenatal care provision and the social and cultural context of some women in LMICs. Antenatal care provision that is theoretically and contextually at odds with local contextual beliefs and experiences is likely to be underused, especially when attendance generates increased personal risks of lost family resources or physical danger during travel, when the promised care is not delivered because of resource constraints, and when women experience covert or overt abuse in care settings.

*Please see later in the article for the Editors' Summary*

## Abstract 2

In a synthesis of 21 qualitative studies representing the views of more than 1,230 women from 15 countries, Kenneth Finlayson and Soo Downe examine the reasons why many women in low- and middle-income countries do not receive adequate antenatal care.

## Editors' Summary

**Background:**

Although maternal deaths worldwide have almost halved since 1990, according to the latest figures, every day roughly 800 women and adolescent girls still die from the complications of pregnancy or childbirth: in 2010, 287,000 women died during or following pregnancy and childbirth, with almost all of these deaths (99%) occurring in low-resource settings. Most maternal deaths are avoidable, as the interventions to prevent or manage the most common complications (severe bleeding, infections, high blood pressure during pregnancy, and unsafe abortion) are well known. Furthermore, many of these complications can be prevented, detected, or treated during antenatal care visits with trained health workers.

**Why Was This Study Done?:**

The World Health Organization (WHO) recommends a minimum of four antenatal visits per pregnancy, but according to WHO figures, between 2005 and 2010 only 53% of pregnant women worldwide attended the recommended four antenatal visits; in low-income countries, this figure was a disappointing 36%. Unfortunately, despite huge international efforts to promote and provide antenatal care, there has been little improvement in these statistics over the past decade. It is therefore important to investigate the reasons for poor antenatal attendance and to seek the views of users of antenatal care. In this study, the researchers combined studies from low- and middle-income countries (LMICs) that included women's views on antenatal care in a meta-synthesis of qualitative studies (qualitative research uses techniques, such as structured interviews, to gather an in-depth understanding of human behaviour, and a meta-synthesis combines and interprets information across studies, contexts, and populations).

**What Did the Researchers Do and Find?:**

The researchers searched several medical, sociological, and psychological databases to find appropriate qualitative studies published between January 1980 and February 2012 that explored the antenatal care experiences, attitudes, and beliefs of women from LMICs who had chosen to access antenatal care late (after 12 weeks' gestation), infrequently (less than four times), or not at all. The researchers included 21 studies (out of the 2,997 initially identified) in their synthesis, representing the views of 1,239 women from 15 countries (Bangladesh, Benin, Cambodia, Gambia, India, Indonesia, Kenya, Lebanon, Mexico, Mozambique, Nepal, Pakistan, South Africa, Tanzania, and Uganda) who were either interviewed directly or gave their opinion as part of a focus group.

The researchers identified three main themes. The first theme reflects women's views that pregnancy is a healthy state and so saw little reason to visit health professionals when they perceived no risk to their well-being—the researchers called this theme, “pregnancy as socially contingent and physiologically healthy.” The second theme relates to women's limited financial resources, so that even when antenatal care was offered free of charge, the cost of transport to get there, the loss of earnings associated with the visit, and the possibility of having to pay for medicines meant that women were unable to attend—the researchers called this theme “resource use and survival in conditions of extreme poverty.” The third theme the researchers identified related to women's views that the antenatal services were inadequate and that the benefits of attending did not outweigh any potential harms. For example, pregnant women who initially recognized the benefits of antenatal care were often disappointed by the lack of resources they found when they got there and, consequently, decided not to return. The researchers called this theme “not getting it right the first time.”

**What Do These Findings Mean?:**

These findings suggest that there may be a misalignment between the principles that underpin the provision of antenatal care and the beliefs and socio-economic contexts of pregnant women in LMICs, meaning that even high-quality antenatal care may not be used by some pregnant women unless their views and concerns are addressed. The themes identified in this meta-synthesis could provide the basis for a new approach to the design and delivery of antenatal care that takes local beliefs and values and resource availability into account. Such programs might help ensure that antenatal care meets pregnant women's expectations and treats them appropriately so that they want to regularly attend antenatal care.

**Additional Information:**

Please access these websites via the online version of this summary at http://dx.doi.org/10.1371/journal.pmed.1001373.

- Wikipedia describes antenatal care (note that Wikipedia is a free online encyclopedia that anyone can edit)
- The World Health Organization has a wealth of information relating to pregnancy, including antenatal care
- The UK National Institute for Health and Clinical Excellence has evidence-based guidelines on antenatal care
- The White Ribbon Alliance for Safe Motherhood has a series of web pages and links relating to respectful maternity care in LMICs
- International Federation of Gynecology and Obstetrics is an international organization with connections to various maternity initiatives in LMICs
- International Confederation of Midwives has details of the Millennium Development Goals relating to maternity care

## Introduction

Recent estimates of global maternal mortality ratios (MMRs) suggest a substantial decline in recent years [1],[2]. However, current rates of decline will still fall well short of meeting Millennium Development Goal 5 (MDG 5): reducing maternal mortality by 75% by 2015 [3]. Data from the World Health Organization (WHO) indicate that in many low- and middle-income countries (LMICs), especially in sub-Saharan Africa, the rate of decline in MMR is less than 1% per year, and in some countries (e.g., South Africa, Nigeria, Mozambique, and Swaziland) rates even appear to be increasing [1],[4]. This slow rate of progress is starkly highlighted in the most recent “Countdown to 2015” report, which found that only nine of the 74 countries with the highest MMRs in the world were on target to achieve MDG 5 [5].

WHO reports and experts in the field consistently highlight the lack of access to local, adequately resourced health care facilities as an important reason for the relatively slow rate of progress towards achieving MDG 5 [6],[7]. Access includes ensuring comprehensive antenatal care coverage for all pregnant women. Recent estimates indicate that the number of women in LMICs attending at least one antenatal appointment increased from 64% in 1990 to 81% in 2009, and those attending four or more times rose from 35% to 51% over the same period [2]. However, major disparities exist within and between continents, between countries, and between urban and rural populations [8]. As with the MMR figures, the rate of progress is slowest in sub-Saharan Africa, where antenatal coverage rates have improved slightly during the last two decades, but the number of women visiting four or more times has remained static, at about 44% [2].

Although the correlation between “inadequate” antenatal care and high maternal mortality is complicated and contentious, it is widely accepted that antenatal care presents opportunities to identify pregnancy risks and, in a broader sense, to monitor and support the general health care of women who may be susceptible to a range of potentially fatal pathologies including HIV, anaemia, malnutrition, tuberculosis, and malaria [5]–[9].

Global implementation of strategies designed to encourage antenatal attendance tend to be based on the assumption that if high-quality services are provided, people will come to them. However, data from quantitative population-level studies suggest that this is not necessarily the case for some groups of pregnant women. Well-documented socio-demographic data indicate that women from relatively poor backgrounds, living in rural areas, and/or with low levels of education are less likely to access antenatal services, even if they are provided [10]–[12]. Other factors, including having a husband with a low level of education, living a long distance from a clinic, and having high parity, have also been identified as barriers [13]–[17]. Similar factors emerge in reviews of barriers to antenatal care in developed countries [18]–[21], which suggests that the issues for women who remain marginalised at local, national, and global levels are much the same.

Based on the results of a WHO antenatal care randomised trial [22], the standard measure of adequate antenatal care delivery is a minimum of four antenatal visits (with the first occurring during the first trimester) for a woman and her foetus, if they are judged to be healthy following a standard risk assessment [23]. Although some authorities, e.g., the US Agency for International Development, have noted the need for woman-centred, individualised, culturally specific programmes [24], the recent *BMJ* Best Practice guidance on routine antenatal care provision lists a wide range of routine screening, testing, and health education topics, with little emphasis on individual concerns and circumstances [25]. Evidence equating risk-focused, low-frequency antenatal care with clinical outcomes in LMICs is limited, but a recent Cochrane review found that population groups in LMICs receiving fewer antenatal visits (4–6) had an increased risk of perinatal mortality and, in particular, stillbirth [26]. The author of a WHO commentary on this review hypothesizes that the excess perinatal loss for women in LMIC settings may be due to inadequate local tailoring of risk assessment, low numbers of staff, and inadequate training [27]. The WHO manual on antenatal care [23] does not specify how antenatal care should be funded, the nature and relevance of staff attitude and training, or what resources should be available at which level of care provision. However, tacit assumptions are likely to include that staff are available and have high levels of communication and interpersonal skills, and that the programme is affordable, otherwise it would be unlikely to function. Despite the findings of the review, and speculation about the components and the effectiveness of the WHO programme, it remains the standard for adequate antenatal care provision.

Given the potential significance of context in mediating whether women access antenatal care, qualitative studies may provide fresh insights into pertinent issues in specific settings. In terms of LMICs, such studies suggest that some women do not attend antenatal facilities because of deeply held cultural beliefs and/or tribal traditions surrounding the nature of pregnancy and childbirth [28],[29]. Qualitative studies can also illuminate the effect of local policies and incentives, such as the use of antenatal clinic cards to guarantee intra-partum hospital access—a controversial practice in a number of African countries because of the potential for discrimination against women who don't have any record of antenatal clinic attendance [30]. However, because of the highly contextualised nature of individual qualitative studies, policy makers often overlook them, and their findings remain outside of global, national, and local health care strategies [31]. Systematic review and synthesis of qualitative studies can generate hypotheses about how successful programmes work, and why unsuccessful programmes fail certain individuals and groups [32]. To address the latter question with regards to inadequate accessing of antenatal care, we planned to locate, analyse, and synthesise qualitative studies exploring the views, beliefs, and experiences of women from LMICs who did not access antenatal care at all, or accessed it inadequately, according to the WHO definition given above. The intention was to develop hypotheses about lack of attendance that could inform policy development, based on a new understanding of why some women still don't access antenatal care, even when it is made available.

### Qualitative Meta-Synthesis Methodology

The emphasis in meta-synthesis is on rigorous study selection and the careful interpretation of data across studies, contexts, and populations. This combination and interpretation of findings from a number of systematically selected studies in a particular subject area shares methodological similarities with its quantitative equivalent, meta-analysis. When meta-synthesis is used to explain or interpret existing knowledge, e.g., alongside meta-analysis, it can be aggregative and deductive [32]. However, when it is exploring fields where there is little prior information, it is undertaken as an inductive method, designed to generate theoretical insights and hypotheses that can be tested in future research [32]. In the latter case, the classic approach is meta-ethnography [33]. As with qualitative research, the direct findings of meta-synthesis are not usually generalisable, but the theoretical insights or hypotheses arising from the synthesis of the included studies should be transferable to other similar settings and contexts [34]–[36]. In meta-synthesis, as in grounded theory, the comprehensiveness of the analysis is determined by the concept of theoretical saturation. Theoretical saturation is reached when new studies do not change the emerging theory or hypothesis, and when a systematic search for disconfirming cases in all the included studies reinforces the theoretical insights. Given the scope and rigour of meta-synthesis reviews, there is greater potential for them to inform practice, influence policy, and underpin strategy than for individual qualitative studies [37],[38].

## Methods

### Search Strategy and Selection Criteria

The search strategy was designed to locate qualitative studies exploring the antenatal care experiences, attitudes, and/or beliefs of women from LMICs who had chosen to access antenatal care late (after 12 wk gestation), infrequently (less than four times), or not at all [7]. We searched for any studies that might include qualitative data, including survey-based studies with open-ended written responses, mixed methods studies, focus groups, and one-to-one interviews. No language restriction was imposed. All electronic searches used keywords covering the main search domains including “antenatal”, “prenatal”, “maternity”, “pregnancy”, “care”, “service”, “provision”, “access”, and “attendance”. The searches were conducted across a range of medical, sociological, and psychological databases (MEDLINE, Embase, PubMed, AMED [Allied and Complementary Medicine Database], BNI [British Nursing Index], CINAHL [Cumulative Index to Nursing and Allied Health Literature], PsycINFO, Wilson Social Science Abstracts), as well as continent-specific databases such as Latindex (Literatura Latino-Americana e do Caribe em Ciências da Saúde) for South American publications and AJOL (African Journals Online) for articles published in Africa. Where possible, we sought to narrow the search to LMICs by incorporating the World Bank's list of low- and middle-income economies in the search terms [39]. Some specific papers were recommended by colleagues, and we hand-searched relevant journals in the departmental and university libraries. Other articles were obtained from reference lists published in identified studies. The initial search included papers published between 1 January 1980 and 31 March 2011. An updated search was completed on 14–15 February 2012, after which the contents pages of relevant journals were reviewed (via Zetoc) as they were published. These updated searches have provided a means to check that the thematic structure and synthesis developed in the primary analysis continue to hold true as new studies are published (“theoretical saturation”).

Both authors reviewed all of the included papers independently, and then reached a final agreement on inclusion by consensus. All of the papers meeting our eligibility criteria were assessed for quality using an appropriate published tool [40]. This tool incorporates a pragmatic grading system [41] and uses an A–D scoring system. The authors determined grades by consensus, and studies scoring C+ or higher were included in the final review (see Table S1).

### Analysis and Synthesis

Our intention was to generate new theoretical insights that could form the basis for hypothesis testing in the future, so we used the meta-ethnographic approach developed by Noblit and Hare [33]. This approach has been used successfully in meta-synthesis studies related to several different health care settings [19],[35],[42]–[44]. It is not restricted to ethnographic studies, as the approach can incorporate the full range of qualitative methods. We began by identifying the findings from one paper and comparing them with the findings from another, to generate a “long list” of emerging concepts. These early concepts were then examined to identify similarities, in a process that is termed “reciprocal translation”. During this process, some concepts were collapsed together to create a parsimonious thematic structure. Each author then reviewed the themes independently to ensure there were no data that were at odds with our analysis and that no data remained unexplained. This stage of the process is analogous to searching for discomfirming data and is termed “refutational translation” in meta-ethnographic studies [33]. The themes were then synthesised into a “line of argument” synthesis—a phrase or statement that summarises the main findings of the study and the theoretical insights that they generate. This synthesis was then used to create a hypothetical model to explain why women fail to make adequate use of antenatal services in LMICs.

### Reflexive Accounting

In qualitative research, the researcher is the instrument of measurement, and the final analysis is a product of the interaction between the researcher and the data. Reflexive accounting allows the reader of the final research product to assess the degree to which the prior views and experiences of the researchers may have influenced design, data collection, and data interpretation in any specific study. In this case, S. D. believed that interpersonal relationships were likely to be critical in mediating antenatal care use, and K. F. believed that whether women accessed care was most likely to be influenced by personal and/or localised socio-economic circumstances. To minimise the effect of these beliefs, both authors were particularly rigorous in looking for refutational data in these specific areas as the analysis progressed.

## Results

**Figure 1:**
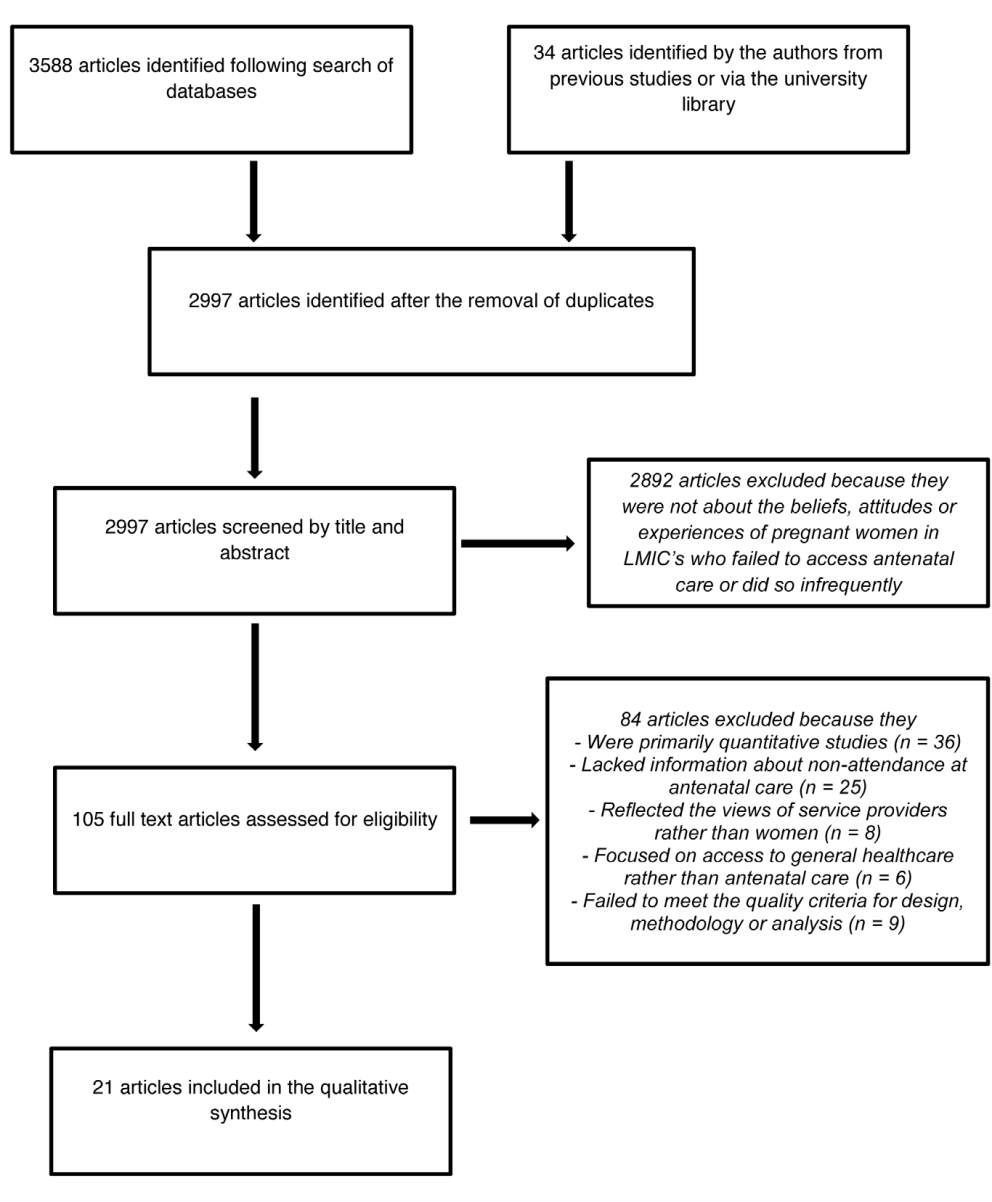
Flow chart summarising search strategy.

Our search to 31 March 2011 generated a total of 3,622 hits, including 625 duplicates, which were removed at this stage. Of the remaining 2,997 articles, 2,892 were excluded by title and abstract because they failed to address the initial selection criteria. Most of the studies removed at this stage were excluded for one of three reasons: (1) they were conducted in high-income countries, (2) they were obviously quantitative, or (3) they were not about access to antenatal care. Of the remaining 105 papers, a further 75 were removed after independent full text review by the authors, largely because they lacked sufficient qualitative data (*n = *36), were based on the experiences of women who attended antenatal services regularly rather those who didn't (*n = *25), reflected the views of service providers rather than the women attending care (*n = *8), or were concerned with access to health care generally as opposed to antenatal care specifically (*n = *6). This left 30 papers that were taken forward for quality assessment. Following independent review, the authors agreed that nine studies failed to meet the quality requirements, leaving 21 that were taken forward for analysis and synthesis (see Figure 1 for details of the selection process). Of the nine studies excluded, three were mixed methods studies with very limited qualitative data, two reported on the views of health care providers with little emphasis on the responses of service users, two presented qualitative information in a quantitative format (frequency of responses), and two failed to meet the quality criteria for design, methodology, and/or analysis. Only one study meeting the inclusion and quality criteria was identified by the updated searches since 31 March 2011 [45], and this was used to check the explanatory power of the final thematic structure, synthesis, and interpretation.

### Description of the Studies

The 21 papers in the final full synthesis represent the views of women from 15 countries (Bangladesh [×2], Benin, Cambodia, Gambia, India, Indonesia, Kenya, Lebanon, Mexico, Mozambique, Nepal, Pakistan, South Africa [×4], Tanzania [×2], and Uganda [×2]) and include data from more than 1,239 participants (minimum 10, maximum 240) who were either interviewed directly or gave their opinion as part of a focus group (see Table S1 for full details of the included studies). Two of the studies utilised a mixed methods approach, and although these studies contained limited qualitative information, the narrative data were pertinent and reasonably well reported. Ten of the 21 studies were conducted in a rural setting, three took place in an exclusively urban environment, and the remaining eight involved both urban and rural settings. The earliest paper was published in 1992 and the most recent in 2011, with the majority (*n = *17) being published within the last ten years. More than half of the included papers (*n = *12) were published within the last three years, which suggests an upswing in interest in this area of research (see Table 1 for a summary of included papers).

**Table 1:** Summary of included studies.

Authors [Reference]	Year of Publication	Country	Location—Type of Region	Number of Participants	Method Used	Quality Grading
Abrahams et al. [46]	2001	South Africa	Cape Town—semi-urban	32	Interviews	C+
Myer and Harrison [47]	2003	South Africa	Hlabisa district—rural	29	Interviews	B
Pretorius and Greeff [48]	2004	South Africa	Mafikeng-Mmbatho districts—rural	18	Interviews	C+
Mrisho et al. [49]	2009	Tanzania	Lindi and Tandahimba districts—rural	58	Focus groups	B
Matsuoka et al. [50]	2010	Cambodia	Kampong and Cham provinces—rural	66	Interviews and focus groups	B
Choudhury and Ahmed [51]	2011	Bangladesh	Rangpur and Kurigram districts—rural	20	Interviews	C+
Chapman [52]	2003	Mozambique	Vila-Gondola—semi-urban	83	Interviews	A
Grossmann-Kendall et al. [53]	2001	Benin	Cotonou and Ouidah districts—urban and rural	19	Interviews	C+
Ndyomugyenyi et al. [54]	1998	Uganda	Kigorobya sub-country—rural	80–120^a^	Focus groups	C+
Gcaba and Brookes [55]	1992	South Africa	Durban—urban	10	Interviews	B
Atuyambe et al. [56]	2009	Uganda	Wakiso district—rural	92	Focus groups	B
Stokes et al. [57]	2008	Gambia	Kiang West district—rural	83	Interviews and focus groups	C+
Griffiths and Stephenson [58]	2001	India	Pune and Mumbai—mix of urban and rural at each location	45	Interviews	B
Simkhada et al. [59]	2010	Nepal	Kathmandu area—semi-urban and rural	30	Interviews	B
Titaley et al. [60]	2010	Indonesia	Garut, Sukabumi, and Ciamis districts, West Java—semi-urban and rural	119	Interviews and focus groups	B
Family Care International [61]	2003	Kenya	Homabay and Migori districts—mix of urban and rural in each	27–47^a^	Interviews and focus groups	B
Tinoco-Ojanguren et al. [62]	2008	Mexico	Chiapas—mix of urban and rural	16	Interviews	C+
Mumtaz and Salway [63]	2007	Pakistan	Punjab—rural	39–55^a^	Interviews and focus groups	B
Chowdhury et al. [64]	2003	Bangladesh	Dhaka and Upazila—urban and rural	16	Interviews	B
Mubyazi et al. [65]	2010	Tanzania	Mkuranaga and Mufinidi districts—both rural	240	Interviews and focus groups	B+
Kabakian-Khasholian et al. [66]	2000	Lebanon	Bekaa, Akkar, and Beirut—rural, semi-rural, and urban	117	Interviews	C+

a A range is given for these studies, as the authors list the number of focus groups conducted, with a minimum and maximum number of participants; e.g., ten focus groups with 8–12 participants.

### Description of the Themes

The emerging concepts and themes are summarised in Table 2. We identified a total of seven emerging themes and three final themes (summarised below), two of which relate specifically to initial attendance at antenatal facilities, and a further, service-oriented, theme relating to maintaining attendance.

**Table 2:** Summary of themes.

Initial Concepts (Findings from Primary Papers)	Relevant Papers (References)	Emerging Themes	Final Themes
Awareness of signs/symptoms of pregnancy	46–51	**Pregnancy awareness and disclosure**—awareness of signs and symptoms of pregnancy; cultural reasons for keeping pregnancy secret	**Pregnancy as socially contingent and physiologically healthy**—pregnancy as a normal life event—only attend antenatal care when sick; lack of awareness of pregnancy indicators; lack of understanding of antenatal care benefits; embarrassment; cultural and supernatural implications of pregnancy disclosure; preference for traditional healers and medicines (including cost savings)
Cultural reasons for keeping pregnancy secret	46,48,49,52–57		
Don't recognise/understand Western approaches to health care	46,47,54,56,58,60	**Resistance to risk-averse care models—**don't recognise/understand Western approaches to health care; lack of perceived benefits; pregnancy as a normal life event; reliance on traditional/alternative antenatal practices; influence of family members	
Lack of perceived benefits of attendance	46–48,51,56,58,59,62–64		
Pregnancy as a normal life event	46,50–54,56,58–61,66		
Reliance on traditional/alternative antenatal practices	50,52,54,58,60–62		
Influence of family members	50,51,62–64		
Costs (direct and indirect)	46,49,50–56,58–66	**Prioritising limited resources for basic survival—**costs (direct and indirect); laziness	**Resource use and survival in conditions of extreme poverty—**costs (direct and indirect), transport, and distance; time off work and child care—may be made to wait several hours; inadequate infrastructure (especially in rural areas); potential for accident/attack en route
Laziness	46,47,49,62		
Lack of transport and distance to clinic	46,48,50,54–56,58,60,65	**Difficult and dangerous travel** —lack of transport and distance to clinic; inadequate infrastructure	
Inadequate infrastructure	48,49,55,58,60–62,64		
Lack of staff/medicine/care at clinic	49,50,54,56,58,65	**Attending clinics is not worth the effort—**lack of staff/medicine/care at clinic; waiting times at clinic	**Not getting it right the first time—**poor staff attitude; inflexibility of antenatal care services; issuing of cards for delivery at a hospital (women don't return) and staff giving card holders preferential treatment; few, poorly trained staff; lack of facilities; lack of medicines
Waiting times at clinic	46,48–50,52,		
Attendance only to get a card (for hospital delivery)	46,47,50,52,61	**Locally determined rules of access—**attendance only to get a card; inflexible booking systems	
Inflexible booking systems	46,63		
Poor staff attitude	46,48–51,53,55–57,62,65,66	**Insensitivity, disrespect, and abuse** —poor staff attitude; embarrassment	
Embarrassment (about examination or inability to pay)	46,49,56,65,		

### Theme One: Pregnancy as Socially Contingent and Physiologically Healthy

This theme incorporates two concepts (highlighted below) that emphasise some of the cultural and contextual nuances associated with pregnancy. Many women in these studies described pregnancy as a healthy physical state and saw little reason to visit health professionals when there was no perceived threat to their well-being. In some cultures this reluctance to engage with antenatal services was further compounded by a belief that pregnancy disclosure could lead to unwanted religious or spiritual complications.

#### Pregnancy awareness and disclosure: “It's better to wait, to see if you really are pregnant”

For many respondents, traditional or cultural beliefs dictated that they should wait until they had missed several periods before confirming a pregnancy [46]–[51].

*Sometimes it's difficult to tell that you are pregnant. Some people have irregular periods, they miss periods for months only to find they are not pregnant, so it is better to wait, to see if you are really pregnant.* [Pregnant woman, rural South Africa] [47]

This belief limited early accessing of care. Even when women suspected they were pregnant, the motivation to visit an antenatal clinic was often superseded by cultural and superstitious beliefs about pregnancy disclosure [52]–[63]. In rural Pakistan, the shame (*sharam*) associated with pregnancy, because of the obvious relationship with sexual activity, meant women were less willing to be seen in public places [63]. The shame of being pregnant and the subsequent reluctance to be seen in public was also a factor for pregnant teenagers in Uganda [56].

In other parts of Africa and South East Asia, the potential to be “cursed” by evil spirits or jealous or vindictive contemporaries had a detrimental effect on pregnancy disclosure [52],[53],[55],[64]. One South African woman who had recently experienced a neonatal death explained her loss in the following manner:

*I think my boyfriend's previous girlfriends were jealous of my pregnancy and they bewitched me.* [55]

These kinds of beliefs appeared to be relatively common in rural communities and discouraged women from visiting public places, especially antenatal clinics, where a visit would be perceived as a public declaration of pregnancy.

#### Resistance to risk-averse care models: “What is the point in going for a check-up in a healthy condition?”

In many of the studies, women reported that they didn't feel the need to seek professional care when there was nothing wrong with their pregnancy [46]–[49],[52]–[54],[58],[63],[64].

*As no-one expects to be sick during pregnancy, visiting the centre for a check-up is not necessary. What is the point in going for a check-up in a healthy condition?* [Non-user of antenatal care services, rural Bangladesh] [64]

Pregnancy was viewed as a normal life event rather than a medical condition requiring professional monitoring and supervision. This was especially true for multiparous women who had experienced one or more healthy pregnancies [50],[54],[58].

*If a woman has always delivered well, she does not see the need for antenatal care attendance or visiting the health unit to deliver.* [Pregnant woman, rural Uganda] [54]

In some hierarchical cultures the decision to engage with antenatal services was made by tribal elders, husbands, mothers-in-law, or senior family members rather than the women themselves [50],[56],[59],[62]–[64]. Findings from a Nepalese study highlight the central role played by the mother-in-law when it came to making decisions about whether to go for antenatal care.

*My mother-in-law doesn't help. It might be due to her past experiences. She used to do all the work by herself during her time of pregnancy, so she wants me to do the same. I have lots of work here at home so I don't go for [antenatal care] check-ups.* [Non-user of antenatal care services, rural Nepal] [59]

### Theme Two: Resource Use and Survival in Conditions of Extreme Poverty

All of the studies were conducted in populations affected by poverty, and our findings suggest that, in such circumstances, limited personal resources were often directed towards immediate survival needs rather than specific pregnancy-related concerns. Even when antenatal care was offered free of charge, the cost of transport (sometimes across difficult or dangerous terrain), the loss of women's labour to the family, and the possibility of having to pay for additional medicines rendered attendance impossible.

#### Using resources for health care or basic survival: “If there is no money, we can't go”

In virtually all of the identified studies [46],[49],[50]–[56],[58]–[66], the costs (both direct and indirect) of visiting antenatal facilities were viewed as a significant factor in restricting or inhibiting access to antenatal care:

*It is good to go to the doctor during pregnancy, but if there is no money we can't go. I wanted to go but I didn't have the money to pay.* [Limited user of antenatal care services, Mumbai, India] [58]

Even in countries offering free access to antenatal care, the unanticipated costs of paying for drugs, tests, and medical cards placed an additional strain on limited family finances.

*The reason I did not go back there [to the antenatal clinic] is because my husband only buys what he wants when he is given the prescription. For example, when there are three things prescribed he buys only two. So, why should I take the trouble to go there for nothing? If I go to the clinic every month, he will just ask how much I think he earns to be able to buy so many medications for me.* [Limited user of antenatal care services, Benin] [53]

The indirect costs of getting to and from antenatal facilities were highlighted consistently in the included studies, especially those conducted in rural areas [46],[48],[50],[54]–[56],[58],[60]. The prohibitive costs of taxi and bus fares prevented some women from visiting antenatal clinics, and, in cases of extreme poverty, even the most basic forms of transport came at an unaffordable price.

*When I was pregnant what prevented me from seeking healthcare was lack of transport money because my legs were a problem. I used to live far away in the hills and I could not ask anyone to take me on a bicycle because I would be asked for money.* [Adolescent limited user of antenatal care services, rural Uganda] [56]

Some of the respondents' accounts indicated that the need for women to contribute to relatively meagre household resources was more than simply a useful contribution. It was perceived to be crucial for survival, especially at significant times in the farming cycle:

*When I had a third pregnancy, it was harvest season. So I wanted to help my husband, even during the pregnancy.* [Non-user of antenatal care services, rural Cambodia] [50]

#### Difficult and dangerous travel: “It is so far and the road condition is too bad”

Many of the studies included in this synthesis were conducted in predominantly rural areas with relatively basic transport networks. For pregnant women living in towns and villages without community health care facilities, the need to journey to distant locations to receive antenatal care presented travelling difficulties, which they were unwilling or unable to overcome [46],[47],[50],[52],[58],[60],[62].

*I never visited the health centre to check my pregnancy because it is so far and the road condition is too bad.* [Non-user of antenatal care services, rural Cambodia] [50]

Even in situations where women were prepared to make lengthy journeys on foot, sometimes walking for three to four hours, the associated risks and challenges occasionally prevented them from doing so. In parts of Africa, the prospect of being attacked by wild animals or aggressive men deterred women from making these journeys, whilst in South East Asia, the deterioration of the roads during the rainy season had a similar detrimental effect. This suggests that the barriers were not just nonexistent or expensive transportation, or inadequate roads, but also the fear of physical harm, which outweighed any benefits that might be gained from antenatal care:

*It is really hard when it is raining. We are afraid we will fall over because the road is so slippery and we are pregnant. The health centre is far and you can see that the road is poor.* [Limited user of antenatal care services, West Java, Indonesia] [60]

### Theme Three: Not Getting It Right the First Time

Given the very real and critical issues of how women perceive pregnancy, and of the economic and physical sacrifice needed from the woman and her family to get her to a central antenatal clinic, it is crucial that the services she receives when she gets there are “fit for purpose”, and that the benefits are perceived to outweigh potential harms. Unfortunately, for many of the women included in this review, this was not the case.

#### Attending clinics is not worth the effort: “It's better to go to the TBA [traditional birth attendant]”

The relatively poor economic circumstances of the countries included in this study meant that clinics were often severely under-resourced. Pregnant women who initially recognised the benefits of antenatal care and who made the often significant financial and personal sacrifices to visit health care facilities were often disappointed by the lack of resources they found when they finally got there. As a consequence, they made the decision not to return [48]–[50],[54],[56],[58],[65].

*I don't visit the health centre for antenatal care because the health centre doesn't have enough medical equipment. When we have a problem, all they will probably do is refer us to the referral hospital….* [Non-user of antenatal care services, rural Cambodia] [50]

The amount of time women had to wait to be seen by health professionals, especially after long and difficult journeys, was a common cause of complaint and discouraged some of them from attending again [46],[48],[49],[52],[54],[65]. Pregnant women also complained about the cursory nature of consultations in understaffed clinics and made the decision to revert to more traditional forms of antenatal care.

*They just touch your abdomen, it's better to go to the TBA [traditional birth attendant] because the TBA examines the mother and tells her how the baby is lying in her stomach.* [Pregnant woman, rural Uganda] [54]

#### Locally determined rules of access: “If you do not have a card, they will not accept you”

Our findings suggest that in a number of cases, particularly in sub-Saharan Africa, the practice of giving antenatal cards to women attending the clinic is being poorly managed and is having a detrimental effect on continued access. Some health care providers use the clinic card as “a passport” and refuse to admit labouring women to a clinic or hospital if they do not have one [46],[49],[52],[53],[65]. This kind of negative reinforcement has created a situation in which pregnant women visit an antenatal facility only once—to get a “clinic card”.

*I am just afraid of being denied services when I need them, so one must just go [to antenatal care] to get the [clinic] card. If you do not have a card, they will not accept you when there is a problem…otherwise we could just rest at home.* [Woman in ninth month of pregnancy, rural Tanzania] [65]

#### Disrespect and abuse: “They don't care for patients”

One of the most common reasons given for delaying or restricting antenatal visits was the poor attitude of staff at health care facilities [46],[48],[50],[51],[53],[56],[62],[63],[65],[66]. Findings from countries in Africa, Asia, and South America highlight insensitivity, rudeness, humiliation, neglect, abuse, and even physical violence by health centre staff as key factors in limiting women's accessing of antenatal care. Sometimes the poor attitude of health care providers was described by what they failed to do, as recounted by a young woman in Uganda:

*They [health care workers at an antenatal clinic] don't care for patients, for example when you go in the morning they will ask you “at your home don't you sleep”. When you go at lunch time they will ask you whether at your place you don't take lunch. And when you go in the evening they will tell you they have closed up.* [56]

Authors also reported that women felt intimidated because of the potential for abuse:

*When you see the health agent yelling at women who are not feeling well, you are afraid of telling them what is wrong with you too….* [Pregnant woman, Benin] [53]

In other contexts, women recounted being punished or humiliated because of some perceived minor misdemeanour:

*If you arrive late at the clinic, the staff rebukes and punishes you with a fine or they order you to clean the floor or sweep the surroundings.* [Limited user of antenatal care services, rural Tanzania] [65]

In all of these examples, women reported feeling reluctant to return for another appointment, and some reverted to more traditional forms of antenatal care as a consequence.

### Line-of-Argument Synthesis

Antenatal care provision that is based on a concept of pregnancy as a potentially risky biomedical state, and that, as a consequence, provides mechanisms focused mainly on surveillance in more or less centralised locations, is contextually at odds with the theories, beliefs, and socio-economic situations of pregnant women and their families in a range of LMICs. This situation is compounded when accessing services presents additional risks to women and their families, in terms of lost labour or income, or physical danger; when the promised care is not delivered because of resource constraints; and when women experience covert or overt abuse in care settings.

### Hypothesis Based on the Findings

**Figure 2:**
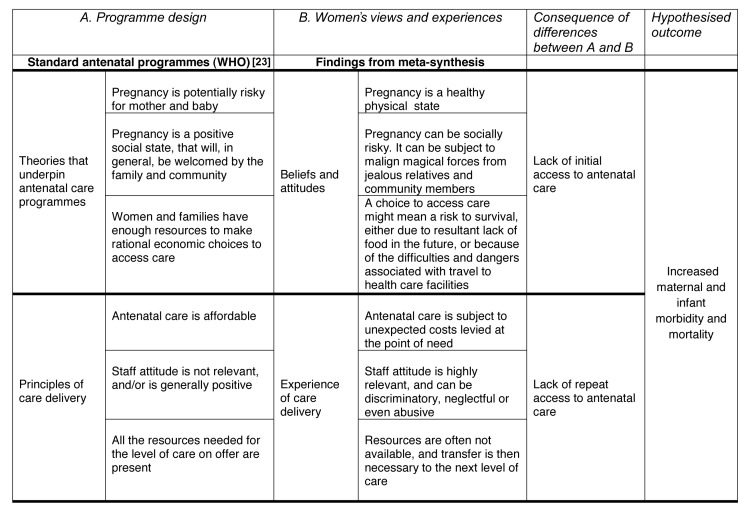
Hypothetical model of inadequate access to antenatal care in low and middle income countries.

Following the claim by Pawson [67] that “programmes are theory incarnate”, our data can illuminate the potential inconsistencies between theories underpinning antenatal care programmes based on the WHO antenatal care model [23] and the themes that underpin the beliefs, actions, and experiences (the local context) of those to whom these programmes are targeted (see Figure 2). We hypothesize that the dissonance between these two frames of reference might explain the lack of initial attendance at antenatal clinics, as described in the first row of Figure 2. The second row of the figure illustrates a second misalignment, this time between the principles assumed to underpin antenatal care provision, and the experiences of women who use them. We hypothesize that this misalignment may explain the lack of return visits for antenatal care after the first encounter.

### Testing for Theoretical Saturation

The data from the one paper [45] we identified after the end of our formal search phase in March 2011 can be incorporated into our explanatory model, suggesting theoretical saturation. We would argue that future studies should therefore focus on testing our hypothesis, and designing specific solutions to the problem of inadequate attendance building on this summary of all the relevant qualitative data to date. This approach would avoid the problem of “analytic interruptus” described by Statham in relation to the continual reproduction of single-site qualitative studies with no attempt to translate the emerging theoretical insights into action [68].

## Discussion

Some of the issues identified by this meta-synthesis are common to other areas of maternity care and health care in general. At the family level, these include lack of household resources, especially when faced with the problem of formal and informal payment or services [69],[70], and the problems inherent in travel to centralised health care services [46],[51]–[54],[71]. Restricted autonomy for women has been identified as a factor underpinning inability to make personal decisions about health service use [72], and this factor is one of the underlying elements of the “three delays” hypothesis relating to lack of accessing of emergency care in labour [73]. There is also an increasing recognition of the problem of human rights abuses in health care in general [74]–[76].

From a theoretical perspective our findings suggest the hypothesis that, in certain contexts, there may be a misalignment between the theories that underpin the provision of antenatal care and the beliefs and socio-economic contexts of women who access services irregularly or not at all. The dissonance between these two frames of reference might explain the lack of initial access to antenatal care. A second disparity, this time between the nature of antenatal provision and the expectations of the women who use the services, may explain the lack of continued engagement. We are not aware of previous studies that have integrated these factors into an analysis of antenatal care use based on women's views and experiences, or that have identified pregnancy as a socially risky but physically healthy state. Minimising social stigma and risk requires care provision that is discrete and certainly not provided in public places subject to long queues for services. Strategies incorporating culturally appropriate understandings of maternity care tailored to individual communities are rare, but participatory programmes where local women and community leaders are actively engaged in the planning of local antenatal services have been shown to be effective in increasing antenatal coverage and reducing maternal and infant mortality [77]. This approach is well illustrated by a recent Cambodian study, which showed a 22% increase in antenatal attendance when service users and influential stakeholders became involved in the planning of community maternity services [78].

From a socio-economic perspective our findings suggest that, even in situations where women recognise the benefits of antenatal care and are willing, in principle, to attend, the physical barriers and even physical risks of getting to and from the clinic, coupled with the potential loss of crucial family resources, can be prohibitive. Even if services are free (with no covert point-of-care costs) and safe transport systems are provided, taking women from essential farming duties on long trips to and from central clinics might, at the extreme, still present a risk to family food supplies. In this case, the benefits of antenatal care must weigh strongly in the balance for service users before uptake will increase. Also, even where women do have a degree of personal autonomy, those who see pregnancy as a healthy state, but as socially risky, are still unlikely to value and attend programmes that expose their pregnant state, and that are largely focused on identifying and averting risk. This is especially pertinent when both the direct and opportunity costs are high, travel to central locations is difficult and dangerous, and the services they receive are of poor quality and overtly or covertly abusive.

Projects designed to incentivise pregnant women to attend antenatal care have been implemented successfully in some LMICs. The Janani Suraksha Yojana cash transfer programme in India, where women are paid a small amount to attend antenatal care and give birth in a recognised health care facility, has had a significant effect on antenatal attendance and subsequent levels of neonatal and perinatal mortality [79]. An alternative, transport-based project in eastern Uganda, where local motorcycle riders were contracted to take women to and from antenatal clinics throughout their pregnancy, also showed a significant increase in antenatal attendance [80]. However, doubts remain about the practicality and sustainability of these kinds of initiatives, and, as our findings illustrate, many pregnant women remain unconvinced by the focus and quality of antenatal programmes, and seem unlikely to make full use of antenatal facilities unless care quality is improved.

Given that data were not available from every region of every LMIC, it is possible that our line-of-argument synthesis, and our model, do not apply to all contexts in which antenatal care is underused. However, the comprehensiveness of our analysis is reinforced by evidence of theoretical saturation, both from our refutational analysis, and from the paper [45] published after the end of our formal search phase in March 2011. Our hypothetical model can explain the findings of this study, including the influence of cultural beliefs and lack of respect from health care professionals. In addition, the findings of the meta-synthesis are similar to those of a parallel review of women's accounts of non-accessing or limited accessing of antenatal care in resource-rich countries [19]. Given the range of countries that were represented in the meta-synthesis, and the date range of the publications (over nearly two decades), the issues seem to be universal and persistent.

We hope that our synthesis illustrates the need to move from small repeated studies of the same problem in local contexts towards a more comprehensive understanding of the potential dissonance between context and service delivery mechanism across all of these settings. A more thorough evaluation using the realist review approach could test this hypothesis, and contribute towards a more detailed understanding of this issue [81]. This could provide the basis for a new approach to the design and delivery of antenatal care, founded on a careful analysis of distinctive local beliefs, values, and resource availability. Such an approach could identify a way of moving services away from broad population-based solutions, towards new service designs based on what works, for whom, in what circumstances [81].

### Conclusion

Despite moderate success in reducing MMRs and increasing antenatal care coverage, the global targets associated with MDG 5 seem unlikely to be attained by 2015, especially in many LMICs. So far, practical initiatives to address these issues have tended to adopt centralised, public provision of antenatal care based on utilitarian strategies designed to minimise clinical risk. This approach benefits some women and infants, but it marginalises others, as the programme design does not take into account necessary survival decisions, beliefs, attitudes, or cultural theories that may be intrinsic to the local context. Measures designed to sustain and maintain access in LMICs are likely to be more effective when policy makers and service providers align their programmes with the theoretical and philosophical constructs and the severe practical constraints that underpin the local community context. Such programmes must ensure that, once they access services, all pregnant women are treated with dignity, respect, and compassion. If programme delivery is not aligned with local contexts in this way, the findings from this meta-synthesis suggest, even the best and most physically accessible services may remain underused by some local pregnant women.

## Supporting Information

Table S1 **Assessment of quality of included studies.**
(RTF)

## Abbreviations

LMIC: low- and middle-income country
MDG 5: Millennium Development Goal 5
MMR: maternal mortality ratio
WHO: World Health Organization
