# Supplementary material for: Why Do Women Not Use Antenatal Services in Low- and Middle-Income Countries? A Meta-Synthesis of Qualitative Studies
Source: PLoS Med. 2013 Jan 22;10(1):e1001373. doi: 10.1371/journal.pmed.1001373 (PMC3551970; doi:10.1371/journal.pmed.1001373)
Supplement: Table S1 — Assessment of quality of included studies. (RTF) [file pmed.1001373.s001.rtf]

Table S1: Assessment of Quality of Included Studies 			
Author/date	Abrahams Jewkes & Mvo
South Africa
2001	Myer & Harrison
South Africa
2003	Pretorius & Greeff
South Africa
2004	Mrisho et al
Tanzania
2009	
Reference	46	47	48 (Mixed Methods)	49	
Clear statement of aims appropriate to question
	Yes – To investigate the health seeking beliefs of pregnant women 	Yes – factors affecting ANC use as part of a broader study looking at screening for syphilis	Yes – to determine the composition of the infrastructure of ANC services in the Mafikeng-Mmabatho area of RSA, and to explore women's perceptions of these services.  	Yes – women's experiences of using ANC and post natal services within the broader context of an ethnographic study	
Literature review thorough and appropriate	Yes - within South African context	Brief summary	Yes –  brief 	Yes 	
Theoretical perspective and design clear and appropriate	Not clear and slightly confusing design	Not discussed 	Mixed methods study – theoretical perspective not  discussed  	No - Clear design but little about theoretical perspective	
Sampling strategy explained and appropriate	Limited details about sampling	Not discussed	Yes - Qualitative arm used retrospective purposive sampling dividing women into ANC attenders and non-attenders. 	No 	
Data collection described and justified	Yes	Brief details	Yes – detailed description (3 phases – final phase qualitative)	Yes 	
Analysis adequately described	Not discussed 	Yes – conceptual analysis	Yes – Coding based on a version of grounded theory. Guba's framework and modelling used 	Yes – brief description – triangulation, coding, identification of themes and discrepancies	
Findings reflect data	 Yes	Yes	Yes 	Yes 	
Researcher reflexivity demonstrated 	No	No	 researcher involvement is discussed though not reflexively	Not discussed	
Study carried out ethically	Yes	Yes	not discussed.	Yes	
Transferability addressed (setting & context adequately described	 Yes -specific to post apartheid South Africa	Yes – briefly discussed in context	Yes =-Setting described though context not fully addressed. 	Yes 	
Relevance and usefulness addressed	Yes- within context	Yes – appropriate recommendations made	Yes - Recommendations made 	Yes 	
QUALITY RATING	C+	B	C+ 	B	


Key to quality rating:- A  No or few flaws. The study credibility, transferability, dependability, and confirmability is high; B – Some flaws, unlikely to affect the credibility, transferability, dependability, and/or confirmability of the study; C – Some flaws which may affect the credibility, transferability, dependability, and/or confirmability of the study; D – Significant flaws which are very likely to affect the credibility, transferability, dependability, and/or confirmability of the study.


Author/date	Matsuoka et al
Cambodia
2010	Choudhury & Ahmed

Bangladesh

2011	Chapman, R
Mozambique
2003	Grossmann-Kendall et al
Benin
2001	
Reference	50	51	52	53	
Clear statement of aims appropriate to question
	Yes- to identify the underlying causes of Cambodian women's non-use of maternal health services provided by skilled birth attendants	Yes – to explore maternal care practices amongst a specific group of very poor women in rural Bangladesh (in an area where a govt initiative to encourage access was being implemented	Yes – seeking to find out why 'high risk' women fail to access ANC until late in their pregnancy	Yes – Women's descriptions of the maternity experience in Benin	
Literature review thorough and appropriate	Yes	Yes	Yes – both wide ranging and specific to context	No – Very brief	
Theoretical perspective and design clear and appropriate	Not discussed.	Not discussed 	Yes – socio-cultural perspective within an ethnographic framework	No clear theoretical perspective
	
Sampling strategy explained and appropriate	Yes - Purposive sampling of reproductive age women residing in 6 communities specifically including women who had and who had not used the maternal health services available at government health facilities. 	Not discussed 	Yes – initially systematic and randomised then changed to snowball as 'trust' issues became apparent	Yes -purposive sampling from a larger group who were surveyed. Authors sought a range of experiences	
Data collection described and justified	Yes - Semi-structured interviews with 5 women from each community and five to seven women were recruited to take part in each focus group discussion.	Yes 	Yes	Yes	
Analysis adequately described	Yes - Responses & discussions from the SSIs & FGDs were transcribed & typed, key phrases coded & categorised.	Yes –  a form of coding and thematic analysis	Brief discussion of thematic analysis Construction of taxonomy of possible threats to reproductive health.	No details given	
Findings reflect data	Yes 	Yes – but very limited ANC data	Yes – detailed quotes and ample evidence of participant input	Yes – well supported by quotes but paper only partly covers  antenatal care	
Researcher reflexivity demonstrated 	Not discussed. Reference made to possible selection bias as it was conducted by the community leader.	Not discussed	Yes 	No	
Study carried out ethically	Not  discussed	Yes 	 Not discussed	Not discussed	
Transferability addressed (setting & context adequately described)	Yes	Yes	Yes- extensive	Yes – supported by findings from similar African contexts	
Relevance and usefulness addressed	Yes	Limited	Yes - within cultural context	Recommendations made	
QUALITY RATING	B	C+	A	C+	

Key to quality rating:- A  No or few flaws. The study credibility, transferability, dependability, and confirmability is high; B – Some flaws, unlikely to affect the credibility, transferability, dependability, and/or confirmability of the study; C – Some flaws which may affect the credibility, transferability, dependability, and/or confirmability of the study; D – Significant flaws which are very likely to affect the credibility, transferability, dependability, and/or confirmability of the study


Author/date	Ndyomugyeni et al
Uganda
1998	Gcaba & Brookes
South Africa
1992	Atuyambe et al
Uganda
2009	Stokes, Dumbaya, Owens & Brabin
Gambia

2008	
Reference	54 (Mixed Methods)	55	56	57	
Clear statement of aims appropriate to question
	Yes – reasons for use & non-use of ANC and malaria treatment among pregnant women in rural Uganda	Yes – factors that influence poor utilization of ANC services amongst multiparous women who had previously booked for a hospital delivery	Yes – to explore adolescent health seeking behaviour during pregnancy and early motherhood in order to contribute to health policy formulation and improved access to health care.	Yes – to explore women's perceptions of recognition and disclosure of pregnancy (as well as use of medication) in rural Gambia 	
Literature review thorough and appropriate	Yes - short	Yes – brief but related to specific area identified above	Yes	Yes - Short	
Theoretical perspective and design clear and appropriate	Mixed methods study – theoretical perspective not discussed	Yes – uses the health belief model as a theoretical framework	Not discussed	Yes - Within the context of 'medical ethics'	
Sampling strategy explained and appropriate	Not discussed 	Yes 	Yes 	Yes - Purposive by location then random by population but limited details equating the populations with the villages 	
Data collection described and justified	Brief discussion	Yes	Yes - Extensive description of data collection and management.	Yes 	
Analysis adequately described	Yes -but a little confused: coded data, content analysis, ethnographic summaries & quotes	Not discussed	Yes- transcription, discussion and triangulation. Computer software used to generate key ideas. Latent content analysis technique was used.	Yes 	
Findings reflect data	Yes 	Inadequate 	Yes 	Yes 	
Researcher reflexivity demonstrated 	No	Not discussed	Not discussed 	Not discussed	
Study carried out ethically	Not discussed 	Not discussed	Yes 	Yes	
Transferability addressed (setting & context adequately described)	Yes - Setting and context well described but limitations not discussed 	Yes	Yes	No - Limited	
Relevance and usefulness addressed	Yes - Recommendations made	Yes 	Yes 	Limited	
QUALITY RATING	C+	B	B	C+	


Key to quality rating:- A  No or few flaws. The study credibility, transferability, dependability, and confirmability is high; B – Some flaws, unlikely to affect the credibility, transferability, dependability, and/or confirmability of the study; C – Some flaws which may affect the credibility, transferability, dependability, and/or confirmability of the study; D – Significant flaws which are very likely to affect the credibility, transferability, dependability, and/or confirmability of the study
Author/date	Griffiths & Stephenson
India
2001	Simkhada, Porter & van Teijlingen
Nepal

2010	Titaley, Hunter, Heywood & Dibley
Indonesia

2010	Family Care International
Kenya

2003	
Reference	58	59	60	61	
Clear statement of aims appropriate to question
	Yes - looks specifically at the barriers to ANC utilization among women in an Indian state (comparing rural and urban populations	Yes – access/non-access to ANC from the perspective of the mother-in-law	Yes – as part of a larger study exploring the general use of maternity services in West Java	Yes – within the context of a report rather than a research study	
Literature review thorough and appropriate	Yes - detailed	Yes - Brief 	Yes  	Yes 	
Theoretical perspective and design clear and appropriate	Brief discussion of a theoretical framework based on predisposing factors 	Yes - Design appropriate and rationale explained. 	Not discussed 	No – not appropriate	
Sampling strategy explained and appropriate	Yes - Snowball sampling, stratified according to urban/rural residence 	Yes - Purposive sampling for women via local health workers and social networks. Snowballing??	Yes – purposive sampling by location and population	Yes 	
Data collection described and justified	Yes – contextually explicit	Yes – mixed methods	Yes 	Yes  	
Analysis adequately described	Yes – content analysis, coding & cross case analysis.	Yes 	Yes	Yes – limitations discussed	
Findings reflect data	Yes 	Yes	Yes 	Yes	
Researcher reflexivity demonstrated 	No	Not discussed	Not discussed	No	
Study carried out ethically	Not discussed	Yes 	Yes 	Not discussed	
Transferability addressed (setting & context adequately described)	Yes 	Yes contrasted with other settings where mother-in-law studies have been done 	Yes 	Yes	
Relevance and usefulness addressed	Yes	Yes 	Yes	 Limited	
QUALITY RATING	B	B	B	B	


Key to quality rating:- A  No or few flaws. The study credibility, transferability, dependability, and confirmability is high; B – Some flaws, unlikely to affect the credibility, transferability, dependability, and/or confirmability of the study; C – Some flaws which may affect the credibility, transferability, dependability, and/or confirmability of the study; D – Significant flaws which are very likely to affect the credibility, transferability, dependability, and/or confirmability of the study


Author/date	Tinoco-Ojanguren et al
Mexico
2008	Mumtaz & Salway
Pakistan
2007	Chowdhury, Mahbub & Chowdhury
Bangladesh
2003	Mubyazi et al
Tanzania
2010	Kabakian-Khasholian et al
Lebanon
2000	
Reference	62	63	64 (monograph)	65	66	
Clear statement of aims appropriate to question
	Yes – women's perceptions of risk and/or complications during pregnancy with a population who might not be perceived as health literate.	Yes – How gender norm's and behaviours affect women's ability or desire to engage with maternity services in Pakistan	Yes – to compare and contrast delivery seeking behaviour amongst rural and urban Bangladeshi's from the perspectives of service users and providers. 	Yes – to describe the experience and perceptions of pregnant women about cost and cost barriers for accessing ANC services with emphasis on IPTp in rural Tanzania 	Yes - To understand Lebanese women's views regarding their experiences of pregnancy and childbirth	
Literature review thorough and appropriate	Yes 	Yes 	Yes 	Yes 	Yes  brief	
Theoretical perspective and design clear and appropriate	Yes – within the context of the predominant biomedical model of maternity care	Yes - ethnographic study which also draws on quantitative data. Rationale explained	Little discussion of theory A variety of methods were used to collect data including social mapping, focus groups & interviews	Yes - Design appropriate–a cross sectional study on perceptions and attitudes gathered by FGDs and semi structured exit interviews.	No	
Sampling strategy explained and appropriate	No 	Yes 	Yes – ethnographic mainly purposive sampling	Yes. 	Convenience sampling from 3  different parts of Lebanon (rural, semi-rural and urban)	
Data collection described and justified	Yes 	Yes – fieldwork, observations, interviews, focus groups and case studies	Yes 	Yes – Focus group discussions at village level and exit interviews based on semi-structured interview guides, 	Yes  	
Analysis adequately described	Yes – Thematic analysis based on Miles & Huberman (1994)	Yes – Broadly interpretive with triangulation and respondent validation. Plus survey data analysed with a statistics package	Yes) - Thematic analysis, triangulation of data and 'spot analysis' – where participants interpret the data themselves  	Yes - following transcription by social scientist analysed manually using qualitative content analysis. Themes were then compared and triangulated.	No ?	
Findings reflect data	Yes 	Yes – though mainly descriptive with limited quotes	Yes 	Yes 	Yes 	
Researcher reflexivity demonstrated 	Not discussed	Yes 	Yes 	Not  discussed 	No	
Study carried out ethically	Yes 	Not discussed	Not discussed	Yes.	women gave consent	
Transferability addressed (setting & context adequately described)	Yes 	Yes	Yes	Yes	Yes – but limited in an ANC context	
Relevance and usefulness addressed	Yes 	 Yes, but broad national and international claims based on localised and contextualized data   	Yes	Yes 	 Yes – mainly comparing 'Western' biomedical approach to traditional cultural values	
QUALITY RATING	C+	B	B	B+	C+ (limited ANC perspective)	


Key to quality rating:- A  No or few flaws. The study credibility, transferability, dependability, and confirmability is high; B – Some flaws, unlikely to affect the credibility, transferability, dependability, and/or confirmability of the study; C – Some flaws which may affect the credibility, transferability, dependability, and/or confirmability of the study; D – Significant flaws which are very likely to affect the credibility, transferability, dependability, and/or confirmability of the study
